# A Monoclonal Antibody-Based Copro-ELISA Kit for Canine Echinococcosis to Support the PAHO Effort for Hydatid Disease Control in South America

**DOI:** 10.1371/journal.pntd.0001967

**Published:** 2013-01-10

**Authors:** Noelia Morel, Gabriel Lassabe, Susana Elola, Mauricio Bondad, Silvia Herrera, Carlos Marí, Jerold A. Last, Oscar Jensen, Gualberto Gonzalez-Sapienza

**Affiliations:** 1 Comisión Nacional de Zoonosis, Ministerio de Salud Pública, Montevideo, Uruguay; 2 Cátedra de Inmunología, Facultad de Química, Instituto de Higiene, UDELAR, Montevideo, Uruguay; 3 Instituto Nacional de Salud, Lima, Peru; 4 Dirección General de Salud Pública, Lima, Peru; 5 Pulmonary/Critical Care Medicine, School of Medicine, University of California Davis, Davis, California, United States of America; 6 Departamento de Investigación en Salud, Chubut, Argentina; Agresearch NZ Ltd, New Zealand

## Abstract

Cystic echinococcosis is still a major concern in South America. While some regions show advances in the control of the disease, others have among the highest incidence in the world. To reverse this situation the Pan American Health Organization (PAHO) has launched a regional project on cystic echinococcosis control and surveillance. An early concern of the program was the lack of a standardized diagnostic tool to monitor infection in dogs, a key target of control programs. Under this premise, we have developed a new copro-ELISA test after extensive screening of a large panel of monoclonal antibodies (MAbs) and polyclonal sera, which performs with high standards of sensitivity (92.6%) and specificity (86.4%) as established by necropsy diagnosis of dogs. The key component of the test, MAbEg9 has a convenient IgG isotype and reacts with a periodate-resistant epitope found in high molecular weight components of the worm. Time-course analysis of experimentally infected dogs showed that even animals with a very low number of parasites could be detected as early as day 20 post infection. The test was formulated in a ready-to-use kit format with proven stability of each component for a minimum of 3 months at room temperature. This characteristic facilitates its standardized use and shipping to other laboratories, which was demonstrated by the identical results obtained by two different laboratories in Peru and our own laboratory when a large number of field samples were analyzed independently in a blind fashion.

## Author Summary

Cystic echinococcosis, caused by infection with the larval stage of the *Echinococcus granulosus* tapeworm, is a life-threatening zoonosis of worldwide distribution. The adult worm parasitizes the small intestine of dogs, which become infected after eating offal of an animal contaminated with the parasite, and releases eggs into the environment that can be accidentally ingested by domestic animals or humans, maintaining the life cycle of the parasite. Deworming of dogs is a major component of control programs, and simple and reliable methods are needed to monitor the base-line infection in the canine population. The lack of these tests was recognized as a major obstacle to the PAHO effort to control the disease in South America. This paper describes the development of a diagnostic assay that detects parasite antigens in dog feces. The key component is a monoclonal antibody carefully selected to attain high levels of sensitivity and specificity, which were established with a large panel of field fecal samples obtained from animals diagnosed by necropsy. Several aspects of the long-term stability of the test were optimized to facilitate its shelf-life and transference to other laboratories.

## Introduction

Cystic echinococcosis, caused by infections with *Echinococcus granulosus*, is one of the main zoonoses related to dogs and is affecting most parts of the globe [1]. Different control programs aimed to decrease the impact of the disease have been instrumented in many countries. Although the rate of success has been highly variable, it has become evident that a tight control of dog infections is the key element to arrest the life cycle of the parasite. This has created a demand for reliable diagnostic tests for canine echinococcosis as a tool to obtain epidemiological baseline information and help in the surveillance of hydatid control [2]–[5]. However, accurate diagnosis of dog infection is complex and challenging, and other than careful necropsy of dogs, there is no perfect gold standard [6].

Parasitological examination of eggs is unsafe and not very useful because *E. granulosus* are morphologically difficult to distinguish from other taeniae eggs, and appear late (after the first month) of infection. Traditionally, screening of dogs for *E. granulosus* has been done by arecoline purgation, followed by examination of the purge for parasites by trained personnel. The method is highly specific, but it is tedious, biohazardous, unpopular among dog owners, and its sensitivity is modest, particularly when the parasite burden is low and bowel evacuation is incomplete [7], [8]. As an alternative, different laboratory tests for ante-mortem diagnosis of canine echinococcosis have been developed, including detection of antibodies in serum, Polymerase Chain Reaction (PCR) amplification of parasite DNA and immunological detection of antigens (coproantigens) in fecal samples. Different studies have been carried out to explore the potential use of dog serology to diagnose the disease. However, the systemic immune response to the parasite is poor and consequently the sensitivity attained is also low [4]. Parasite DNA excreted with eggs, proglottids or cells has been detected in fecal specimens by PCR amplification using specific primers derived from mitochondrial DNA [9], [10]. The method has provided the high specificity of PCR, but due to the presence of inhibitors the DNA extraction procedure is cumbersome and the technique requires expensive reagents and specialized laboratories [11]. In addition, the pre-patent period of the infection, when there is no egg production, is a critical time-window that challenges the sensitivity of the test. Introduced at the beginning of the 1990's, the detection of parasite antigens in fecal samples by immunoassays became a widely accepted diagnostic test [12], [13]. The major attractive features of the method include its simplicity, the possibility of detecting parasite components even in the pre-patent period, and the fact that the target antigens (coproantigens) are highly stable. Samples can be collected in 1% of 40% formaldehyde and kept at room temperature for extended periods of time, facilitating the logistics of large-scale studies in remote areas [7], [14]–[16]. The study of a large group of infected dogs necropsied at the end of the pre-patent period demonstrated a superior sensitivity for the copro-ELISA (83%), when compared to copro-PCR (26%) or arecoline purgation (43 and 77% after one or two doses, respectively). Most failures to detect positive infected dogs occurred when the number of worms was less than one hundred [5].

Despite its proven utility and the numerous reports of copro-ELISA developments, the availability of commercial or home-made tests is often a major obstacle to the implementation and evaluation of echinococcosis control programs. The need to overcome this difficulty has been recognized as an early goal of the Southern Cone Sub-Regional Project on Cystic Echinococcosis Control and Surveillance: Argentina, Brazil, Chile and Uruguay, and PAHO (Pan American Health Organization), a joint and collaborative effort to battle the disease in the region. In this framework, we have developed an immunoassay for *E. granulosus* copro-antigen detection under the premise that in addition to performing with high standards of proven sensitivity and specificity, it had to be robust, standardized and developed in a kit format to be available for its use in regional programs for the control of the disease.

## Materials and Methods

### Ethics statements

All activities involving animals were performed with special care to establish high standards of biosafety and assure animal welfare. All protocols were performed according to the International Guiding Principles for Biomedical Research Involving Animals, (CIOMS) and were approved by the Comisión Nacional de Zoonosis and the Research Department of the Ministry of Health of the province of Chubut.

### Experimental and field animals

Nineteen adult dogs, of mixed breeds and sex, were vaccinated against distemper encephalomyelitis, parvovirosis, rabies, leptospirosis, hepatitis and coronavirus (Merial, France), treated orally with praziquantel 5 mg/kg, pyrantel embonate 15 mg/kg and febantel 15 mg/kg (Disper, Uruguay) to eliminate worm parasites, and topically with a solution of fipronil and methoprene (Merial) to arrest the development of ectoparasites. Dogs were purchase from local suppliers and maintained on commercial dog food and water *ad libitum*. Nineteen fecal samples (N1–N19), corresponding to non-infected dogs, were collected two days after deworming. Then, ten dogs (identified as P1–P10) were infected orally with 5,000–150,000 viable (>80%) protoscoleces from ovine fertile hydatid cysts. Two additional dogs (identified as Th1 and Th2) were infected orally with 1 and 3 larvae of *Taenia hydatigena, respectively*. Parasite material was obtained from sheep slaughter in local abattoirs in Paysandú, Uruguay. Stool samples from each dog were collected daily during the experimental period, at the end which (27–30 days post-infection (dpi)), the animals infected with *E. granulosus* were euthanized for necropsy diagnosis by intramuscular injection of acepromazine maleate (0.11 mg/kg) and ketamine (20 mg/kg) (Laboratorio Holliday, Argentina), following by an intravenous overdose of sodium thiopental (Laboratorio Crusur Vet, Uruguay). The dogs infected with *T. hydatigena* were purged at 70 dpi with arecoline bromhydrate (Crescent Chemical Co. Inc., New York, USA), following by treatment with anti-parasitic drugs. Three additional samples from dogs experimentally infected with *E. granulosus* (26 dpi) and two with *T. hydatigena* (about 55 dpi) from previous studies [14], [16] were obtained as a kind gift from Dr. Carlos Carmona. In addition, 85 unwanted dogs from the province of Chubut, Argentina, and 11 unwanted dogs from Junin, Peru, were euthanized following the procedure described above, and the small intestines were removed post mortem, opened longitudinally and examined directly for the presence of parasites following the recommendations of the WHO/OIE [17]. All activities were performed following the local legislation and the International Guiding Principles for Biomedical Research Involving Animals (http://www.cioms.ch/).

### Preparation of fecal samples

Stool samples from each dog were collected daily during the experimental infection period, or from the rectum upon necropsy, in 1% of 40% formaldehyde, phosphate buffered saline (PBS), in a 1∶4 v/v ratio. Samples were vigorously shaken to obtain homogeneous slurries, and were then boiled for 20 min in a water bath, to eliminate their biological risks. After centrifugation for 10 min at 2,200× g the supernatants were aliquoted and kept frozen at −20°C until used.

### Parasite antigens

The parasite antigens were prepared essentially as described by Casaravilla et al. [16]. *E. granulosus* worms were collected from the small intestine of experimentally infected dogs by incubation of small pieces of the open gut in sterile Hank's balanced salt solution (Sigma, San Louis). The recovered worms were counted and extensively washed in Hank's solution containing 200 mg/L of gentamicin, and incubated in RPMI 1640 (Sigma) supplemented with glucose (4 g/L), penicillin (10^5^ IU/L), streptomycin (100 mg/L) and amphotericin B (250 µg/L) (Sigma). The supernatant was collected every 8 h for two days and the excretion/secretion antigens (E/S) concentrated 100× using an Amicon ultrafiltration unit with an YM-10 membrane (Millipore, USA), followed by dialysis against PBS. *E. granulosus* somatic antigen (Sm) was obtained by sonication of adult parasites in PBS supplemented with ULTRA Protease Inhibitor Tablets (Roche, Indianapolis) on ice, centrifugation and filtration (0.22 µm). Antigens from *T. hydatigena* were similarly prepared. Protein content was determined using a BCA kit (Pierce, Rockford, Illinois).

### Polyclonal antibody production

To prepare polyclonal antibodies, 100 µg of Sm or E/S antigens were dissolved in 250 µl of PBS and vigorously mixed with 250 µl of Freund's Complete Adjuvant (Pierce, Illinois) to form a thick emulsion. This emulsion was then injected subcutaneously into several points on the back of New Zealand white rabbits. After 4 and 8 weeks, the animals were immunized intramuscularly with additional doses of 100 µg of antigen emulsified in Freund's Incomplete Adjuvant. Ten days after the final booster the animals were bled. The IgG fraction was prepared by precipitation of the sera with ammonium sulfate, affinity purified on protein G agarose (Amersham, Uppsala, Sweden), and kept at −20°C until used.

### Selection of MAb CPR39

For monoclonal antibody (MAb) preparation, Balb/c mice were primed intraperitonally with 40 µg of *E. granulosus* Sm or E/S antigen in Freund's complete adjuvant, and boosted after 3 and 6 weeks with the corresponding antigen using Freund's incomplete adjuvant. Three days after the last booster mice were sacrificed and the splenocytes fused with SP2/0 cells using standard procedures. Cultures producing MAbs reactive with the corresponding antigen were selected by ELISA. We initially performed a fusion experiment using mice immunized with alternate doses of E/S and Sm antigens, and additional selection in a sandwich format using fecal samples. MAb CPR39 was selected due to its convenient isotype and performance.

### Selection of MAb Eg9

Several fusion experiments were performed to produce a large panel of monoclonal antibodies. The screening of the monoclonal antibodies was initially performed as described before, and then each of the double-positive supernatants (13 clones) was tested in a sandwich format in combination with the different polyclonal antibodies. However, this time instead of using fecal samples from heavily infected dogs, each supernatant was tested against the following: i) a negative sample from a non-infected dog, ii) a sample collected from a low-burden dog at 28 dpi (dog P9, 74worms), iii) a sample from a heavily infected dog taken at an early stage (10 dpi) of the pre-patent period (dog P8, 3459 worms), and iv) a sample from dog Th2 taken at 56 dpi that was positive in the CPR39 copro-ELISA. Out of this complex screening clone MAbEg9, in combination with polyclonal antibody PAbC4 (prepared from a rabbit immunized with E/S), was chosen because this antibody pair produced high signals with samples (ii) and (iii), and low readings with samples (i) and (iv).

### SDS-PAGE and Western blot

Parasite preparations were resolved by SDS-PAGE 12% under reducing conditions and then transferred onto nitrocellulose sheets (Bio-Rad, Hercules, California, USA). The nitrocellulose was blocked with 3% non-fat milk powder in PBS 1 h at 37°C and was incubated for 1 h at 37°C with a 1∶500 dilution of the rabbit anti-sera in PBS containing 0.05% of tween 20 (PBS-T), 3% non-fat milk, or directly with the culture supernatants of the MAbs antibodies. The membranes were then incubated for 1 h at 37°C with alkaline phosphatase-conjugated to anti-rabbit IgG or anti-mouse IgG (Pierce) (diluted 1/2500). After extensive washing, a substrate solution containing 5-Bromo-4-Chloro-3′-Indolylphosphate p-Toluidine salt (BCIP) and Nitro-Blue Tetrazolium Chloride (NBT) was added according to the manufacturer's instructions (Sigma).

### Copro-ELISA assay

Five µg/mL of polyclonal IgG (100 µL/well) (PAb CG10 or PAbC4 for the MAb CPR39 or MabEg9 copro-ELISA, respectively) was dispensed into microtitration plates (Greiner, Germany) and incubated overnight at 4°C. The plates were then blocked with 5% non-fat milk (PBS) and washed with PBS-T. Alternatively, the plates were further treated with PBS, 0.1% bovine serum albumin (BSA), 5% sucrose, 0.02% sodium azide, flapped repeatedly against adsorbent paper, dried in a 40% relative humidity chamber for 4 h, and kept in sealed aluminum foil bags containing adsorbent packets (Sigma) until used. Samples were analyzed in triplicates, after a 1∶2 dilution in PBS; 100 µL/well were incubated for 1 h at room temperature, the wells washed and loaded (100 µL/well) with a 1∶50 dilution of the MAb supernatants and incubated for 1 h at room temperature. Finally, the wells were incubated (1 h) with a 1∶5000 dilution (PBS-T) of a peroxidase conjugated rabbit polyclonal antibody to mouse IgG, carefully washed and developed with 100 µL/well of the substrate solution (0.4 mL of a 6 mg/mL DMSO solution of 3,3′,5,5′-tetramethylbenzidine (TMB), 0.1 mL of 1% H_2_O_2_ in water in a total of 25 mL of 0.1 M citrate acetate buffer pH 5.5) and incubated for 15 min at room temperature with shaking. The enzymatic reaction was stopped after 15–20 min by the addition of 50 µL of 2 M H_2_SO_4_ and the absorbance at 450–600 nm was read in a microtiter plate reader (Multiskan MS, Labsystems). The cut-off for the assay was established by Receiver Operating Characteristic (ROC) analysis [18] using a confidence level of 95% and the SPSS 10.0 software package (SPSS Inc. Illinois, USA) for calculations.

## Results and Discussion

### Parasite extraction and antigen preparation

**Figure 1:**
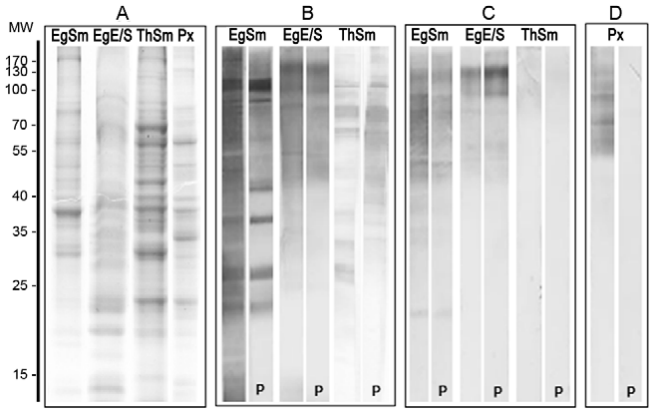
Analysis of parasite antigens by SDS-PAGE and immunoblot. A) Coomassie stained 12% SDS-PAGE of E. granulosus antigens: E/S, excretion/secretion antigen; Sm, somatic antigen; Px, protoescoleces sonicate; and T. hydatigena somatic antigens: ThSm. Molecular markers in kDa are shown on the left. B–D) Immunoblot probed with polyclonal rabbit antibody PAbCGB10 (B), MAbEg9 (C) or E492/G1 (D). The nitrocellulose strips marked with a “P” were pre-treated with periodate to modify the sugar epitopes.

Upon necropsy at 27–30 dpi, 9 of the experimentally infected dogs P1–P10 dogs harbored *E. granulosus* worms. The number of worms recovered from each dog varied widely, from 74 to 8,500, and showed no correlation with the initial number of protoescoleces used for infection. No other parasites were observed in addition to the *E. granulosus* worms. Adult worms (typically less than 1 m long) were recovered from dogs Th1 and Th2 infected with *T. hydatigena*. After careful washing, the recovered parasites were kept in culture to prepare excretion/secretion antigens, or were sonicated as described to obtain somatic antigens. The SDS-PAGE analysis of these antigens is shown in **figure 1A**; a protoescoleces extract was also included as a reference antigen. All preparations possessed a complex composition and the SDS-PAGE profile did not reveal any obvious similarities among the individual components of the different preparation.

### Characterization of antibodies to worm antigens

Initially, the reactivity of polyclonal antibody PAbCGB10 raised against the Sm antigen was characterized, **figure 1B**. There was a strong response to a large number of components of the immunizing antigen, as well as to high molecular weight components of EgE/S and *T hydatigena*. Destruction of sugar epitopes by periodate treatment significantly decreased the reactivity of this antibody with the somatic antigen, as has been observed before [2], [19], but had little effect in the case of the E/S preparation. The marked cross-reactivity of the polyclonal antibodies with E/S components of *T. hydatigena* evidences how difficult it is to obtain a specific assay using these antibodies as reagents. For that reason we concentrated our efforts in the preparation and selection of monoclonal antibodies After initial screening against E/S and Sm and further selection using PAb CGB10 for capture and the hybridoma supernatants for detection, four clones providing the best signal/noise ratios with fecal samples were selected. Among these, clone MAbCPR39, secreting an IgG2b, was chosen.

### MAb CPR39 copro-ELISA

**Figure 2:**
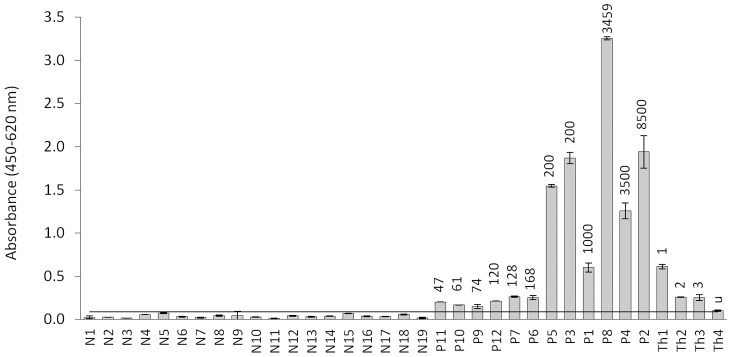
Analysis of coproantigens from non-infected dogs, and *E. granulosus* and *T. hydatigena* experimentally infected animals by CPR39 copro-ELISA. N1–N19, fecal samples taken from non-infected dogs; P1–P12, fecal samples taken from *E. granulosus* infected dogs at the end of the pre-patency period; Th1–Th4, fecal samples collected from *T. hydatigena* infected dogs, at 72, 61, 56, and 40 dpi, respectively, corresponding to the highest values of cross-reactivity during the infection period for each dog. The number of worms recovered from each dog is indicated on top of the columns (u = unknown). The black horizontal line represents the cut-off = average of N-samples+3 standard deviations.

**Figure 3:**
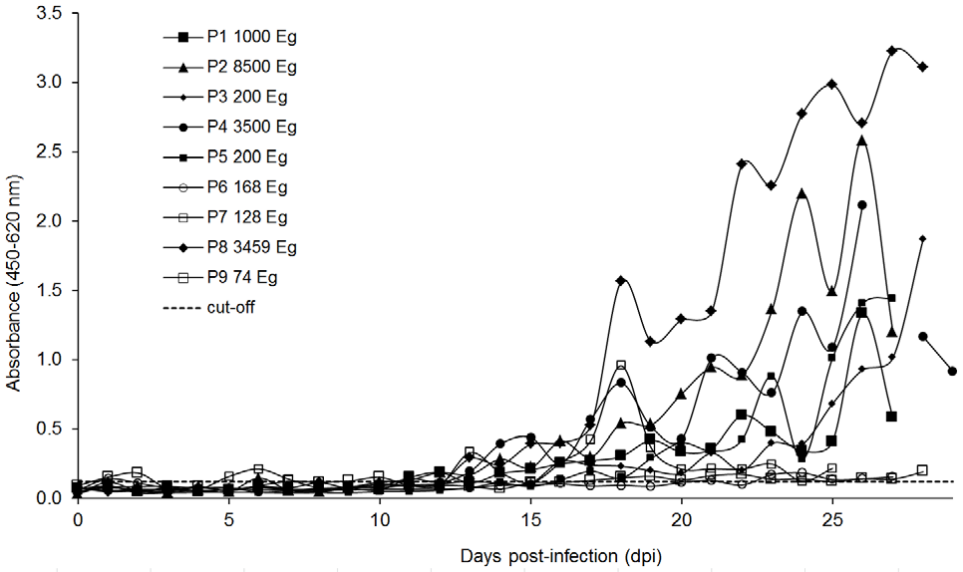
Time course of coproantigen excretion during the experimental infection of dogs assessed with the CPR39 copro-ELISA. The number of parasites at necropsy is shown in the legend; dogs with less than 200 parasites are denoted by open symbols. The dotted line represents the cut-off established by analyzing field samples as described below.

This ELISA was used to examine fecal samples obtained at the end of the infection, days 27–30 for *E. granulosus* or at selected dpi (highest cross-reactivity) for *T. hydatigena* (**figure 2**). The assay performed with negligible reactivity with samples from non-infected dogs and moderate cross-reactivity with *T. hydatigena* infected animals. All *E. granulosus* infected dogs were positive, however, the readouts of the samples corresponding to dogs with small parasite burdens were significantly higher, but too close to the mean value of the negative dogs to give unequivocal results. The assay was then used to study the time course of coproantigen excretion during the experimental infection period (**figure 3**). Using a cut-off derived from the analysis of field samples (see below), we found that dogs with high parasite burdens became positive after two weeks, and in spite of some fluctuations remained so thereafter. On the other hand, dogs with less than 200 worms at necropsy, presented low readouts throughout the experiment, becoming positives only at the end of the experimental infection. Along the time course of the examined period, the dogs harboring *T. hydatigena* worms presented very low values (below the cut-off), showing positive values only in very few days after 40 dpi (not shown).

### MAb EG9 copro-ELISA

Due to the limited sensitivity of the CPR39 copro-ELISA, new antibodies were prepared. The best combination resulted when PAb C4 was used as capture antibody in combination with MAb Eg9. The reactivity of PAbC4 with different parasite antigens in western blot was similar to that of PAbCGB10 (figure 1B) and it is not shown. MAbEg9 is an IgG; its reactivity with different parasite extracts is displayed in figure 1C. The antibody reacted with a large number of the EgSm bands, mostly in the middle to high molecular weight range. Two components of 100 and 160 kDa are the most prominent immunoreactive bands of the E/S antigens, which appear to be also present in the Sm preparation. The cross-reactivity with Th E/S antigens was negligible (as was also the case with protoscoleces antigens, not shown). Treatment with periodate had no effect upon the reactivity of MAbEg9 with any of the antigens, which was unexpected because previous studies have shown that the sugar epitopes have a major role in the immune response against *E. granulosus* coproantigens [2], [19]. To rule out technical artifacts, the efficacy of the periodate treatment was tested in parallel using the monoclonal antibody E492/G1 that defines a sugar epitope highly expressed in *E. granulosus* protoscoleces preparations [20], **figure 1D**.

**Figure 4:**
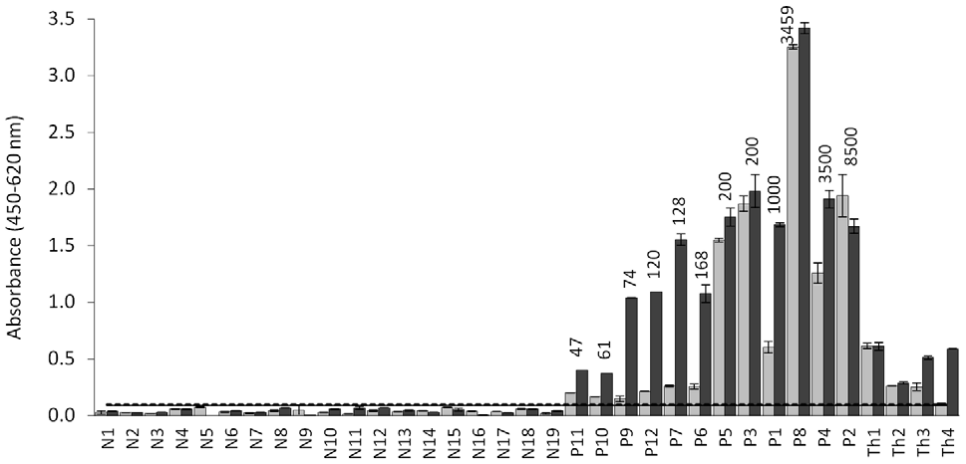
Analysis of coproantigens from non-infected dogs and *E. granulosus* and *T. hydatigena* experimentally infected animals by Eg9 copro-ELISA. Light gray bars, MAbCPR39 assay; gray bars, MAbEg9 assay (the results of the MAbCPR39 copro-ELISA are included for comparison). N1–N19, samples taken from non-infected dogs; P1–P12, samples taken at the end of the pre-patency period; Th1–Th4, fecal samples collected from *T. hydatigena* infected dogs, at 72, 61, 56, and 40 dpi, respectively, corresponding to the highest values of cross-reactivity during the infection period for each dog. The number of worms recovered from each dog is indicated on the columns (u = unknown). The black horizontal line represents the cut-off = average of N-samples (MAbEg9)+3 standard deviations.

**Figure 5:**
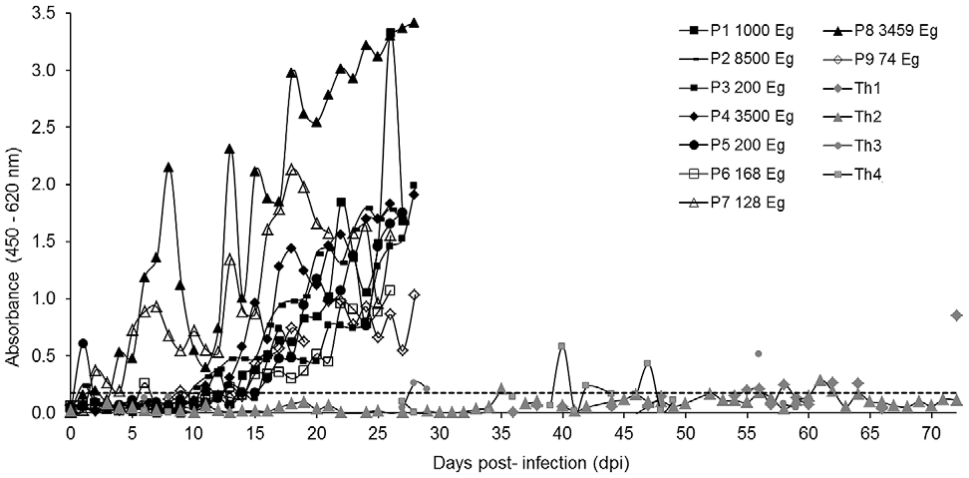
Time course of coproantigen excretion during the experimental infection of dogs measured by Eg9 copro-ELISA. Dogs infected with *E. granulosus* are shown in black, open symbols are used to represent dogs with less than 200 parasites at necropsy. The *T. hydatigena* infected dogs are shown in gray. The dotted line represents the cut-off established by the analysis of field samples as described below.

When the experimentally-infected dog samples were analyzed with the new assay, it was evident that the Eg9 copro-ELISA produced stronger signals with samples from dogs with small numbers of worms, allowing a better discrimination between non-infected and infected dogs (**figure 4**). This is an important improvement because most of the reported tests failed to detect these kind of samples. Cross-reactivity with *T. hydatigena* coproantigens did not seem to be a major problem when the time course of coproantigen excretion was study with the Eg9 copro-ELISA (**figure 5**). Using the cut-off estimated by the receiver operating characteristic (ROC) curves from the analysis of field samples (see below), only on very few occasions and after 40 dpi were some of the samples from *T. hydatigena* infected dogs positive. The Eg9 copro-ELISA allowed an earlier detection of infected dogs. Animals with 200 or more parasites became positive between 12–15 dpi and by dpi 20 all infected dogs were positives and remained so thereafter. Curiously, coproantigens in dogs P7 and P8 samples could be detected very early. This is more remarkable in the case of dog P7 that harbored a rather small number of worms (128) at necropsy, and may be the result of the spontaneous expulsion of worms a few days after infection.

### Analysis of field samples

**Figure 6:**
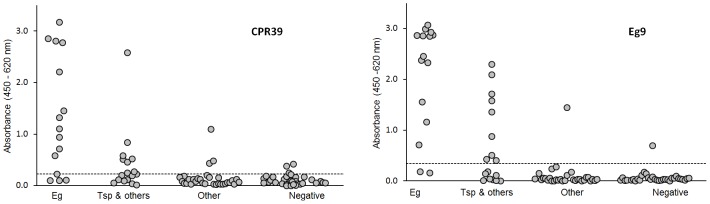
Analysis of field samples with the CPR39 and Eg9 ELISAs. Fecal samples collected from field dogs were grouped according to the parasites they harbored as follows: Eg, *E. granulosus*; Tsp & other, *Taenia spp* plus other helminths; Other, other helminths; Negative, dogs with no helminths. All values were determined in triplicates.

The cut-off and cross-reactivity of the assays were evaluated with field samples collected from 96 unwanted dogs in the provinces of Chubut, Argentina and Junin, Peru. Upon necropsy, 9 of 85 dogs from Chubut, and 6 of 11 dogs from Junin were positive for *E. granulosus*, 48 were infected with other parasites and 33 were found negative, **table 1**. Fecal samples from these dogs were tested using the CPR39 and Eg9 copro-ELISAs and the cut-off selected from receiver operating characteristic (ROC) curves [18] to have a convenient balance between sensitivity and specificity as follows: CPR39 ELISA = 0.224 AU and Eg9 ELISA = 0.340 AU. Using these values, the sensitivity of the tests were 80.0 and 86.6% respectively, which is in agreement with the results obtained with the experimental infection of dogs, **figure 4**. The specificity of the Eg9 test was also superior 86.4% versus 82.7% for the CPR39 ELISA. In addition, the CPR39 test produced readings close to the cut-off for an important number of samples, **figure 6**, which may be problematic for the use of a cut-off internal calibrator, because small inter-assay variations in the reading of the calibrator may have an important effect upon the number of samples that are classified in either category. Actually, if samples that fall between ±10% of the cut-off are considered as undetermined (a common practice), none of the samples analyzed with the Eg9 test would fall in this category, while four of the samples analyzed with the CPR39 would. While it was not possible to obtain an exact counting of parasites in the dogs from the province of Chubut, this was feasible in the case of the animals necropsied in Junin. Although most dogs had a large number of worms, two of them did not, and these two dogs were exaustively examined. We found only 9 *E. granulosus* and 2 *Ascaris lumbricoides*, and 47 *E. granulosus* and very few *Dipylidium caninum* worms in the first and second dogs, respectively. Interestingly, the average absorbance readings of the fecal samples obtained from these animal were 1.16 and 2.99 AU, which represent strong positive results, indicating the sensitivity of the test and confirming the capacity of the assay to detect small numbers of parasites that had been observed in the experimentally infected dogs. In this regard, when the *E. granulosus* positive dogs from the experimental infection are included, the overall sensitivity of the test rises to 92.6%. Despite the fact that MAbEg9 was selected for its reduced cross-reactivity with the ThSm antigen, most of its cross-reactivity was against dogs infected with *T. spp* (mostly *hydatigena*) alone or together with other parasites. Only one dog infected with *T. canis* (1.44 AU) and one of the non-infected dogs (0.694 AU) from the province of Chubut were classified as positive with the Eg9 ELISA, **figure 6**. The relatively high readings of these two samples are unexpected. Based on the results of the experimental infection of dogs and the fact that all other samples (19) from dogs harboring *T. canis* alone or together with *Dipylidium caninum* showed very low readings, a misdiagnosis at necropsy can not be discarded. It is worth noting that cross-reactivity was essentially against Taenia. Despite the fact that we could not identify to species all field samples, the great majority of samples from Chubut and all from Junin corresponded to *T. hydatigena*; hence most false positive results will be related to dogs eating offal.

**Table 1:** Analysis of field samples by necropsy and copro ELISA.

Necropsy results	Total	CPR39+	Eg9+
Eg (and others)	15	12	13
Tsp	8	3	3
Tsp To Dc	3	1	3
Tsp To	6	4	3
To	17	1	1
Dc	10	2	0
To Dc	3	0	0
Tt	1	0	0
Negatives	33	3	1
Sensitivity		80.0%	86.6%
Specificity		82.7%	86.4%

Eg: *E.granulosus*, Tsp: *Taenia spp*, Dc: *Dipylidium caninum*, To: *Toxocara canis*, Tt: *Trichuris trichura*. CPR39+ and Eg9+ indicate samples classified as positive by the respective tests.

### Development of the ELISA test in a kit format and inter-laboratory analysis of blind samples

**Figure 7:**
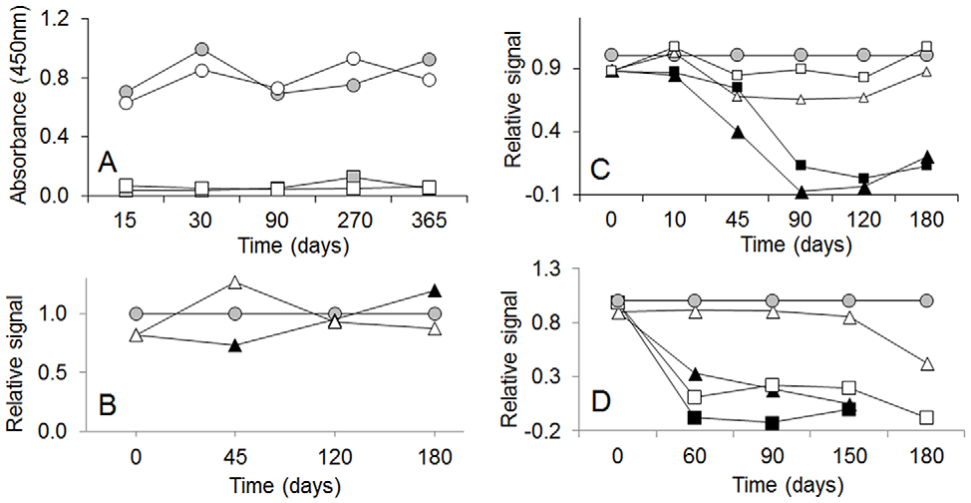
Time-course stability of the copro-ELISA kit components. A) Dried or freshly-coated plates (white and gray symbols, respectively) were tested at different time points by assaying a set of 3 weak-positive and 3 negative samples (average = circles and squares, respectively). B–D) Fresh solutions of the calibrator were tested in triplicate at different time points, using fresh (grey circles) or stored dilutions of the kit reagents kept at room temperature (white) or 37°C (black symbols). All values were normalized with regard to the value obtained with the fresh reagent. B) Stability of the calibrator: triangles, PBS, 5% glycerol, 0.1% Kathon. C) MAb Eg9 stability: squares, PBS-T; triangles, PBS-T, 0.1% Kathon. D) Stability of the peroxidase anti-mouse IgG antibody: squares, PBS, 0.1% BSA; triangles, PBS, 0.1% BSA, 0.1% Kathon, 0.1 mM 3,3′,5,5′-tetramethylbenzidine (TMB).

The Eg9 test was then formulated in a kit format to facilitate its use and transference to other laboratories. Initially, we studied different conditions for coating and drying the plates and found that using sugars as additives during drying have the best effect on the stability of the capture antibody. Trehalose and sucrose showed the best results and the latter was chosen because of its much lower cost. **Figure 7A** shows that dried plates showed similar capacity as fresh ones to discriminate between weak positive and negative samples over a one year period of storage at 4°C. Similar results were obtained after 2 years of storage (not shown). To set up the value of the cut-off an internal standard was prepared by adjusting the dilution of the *E. granulosus* Sm antigen in order to produce a readout equal to the value of the cut-off. The stability of this cut-off calibrator solution and other components of the assay (in their ready-to-use dilution) were tested using different diluent buffers at 4°C, room temperature, and 37°C, and some representative results are shown in **figure 7B–D**. Different formulations of the calibrator solution were stable over a 6 month period, even at 37°C; only the results for the Sm antigen diluted in PBS, 5% glycerol, 0.1% Kathon (Dow Chemicals, Midland, Michigan) are shown. The reactivity of MAb Eg9 started to diminish after a few days at 37°C, but was highly stable over the 6 month period at 4°C (not shown) or at room temperature, **figure 7C**. The most critical component was the secondary antibody. For several different formulations (only two shown in **figure 7D**) all of them rapidly lost the enzymatic activity at 37°C, but using PBS, 0.1% BSA, 0.1% Kathon, 0.1 mM TMB, the conjugate remained stable for up to 150 days at room temperature (or 4°C, not shown). Overall, we can conclude that even at room temperature the kit has a safe shelf life of 3 months, which can surely be extended upon refrigeration, which is a convenient advantage for the shipment and use of the kit in remote places. Due to the availability of commercial ready-to-use TMB substrate solutions, the preparation of this kit component was not pursued in this study. In case of high demand of the kit, it will be necessary to prepare new PAb. In our experience, this is a critical component of the test, but we have selected similarly performing PAbs from a panel of 3 to 4 immunized rabbits, after initial selection with fecal samples from dogs harboring low number of *E. granulosus* parasites and counter selection with samples from *T. hydatigena* infected dogs.

The reproducibility of the results obtained with the copro-ELISA kit and the feasibility of its transference were demonstrated by the analysis of blind samples. To this end, 52 fecal samples collected in the province of Junin, Peru were processed as described, and aliquots were distributed to two laboratories (INS and DIGESA) in Lima, Peru, where the copro-ELISA kit had been transferred, and to our laboratory in Uruguay (Facultad de Química). The samples were independently analyzed by the three laboratories and classified as positive, negative or uncertain if their readout felt within the calibrator absorbance reading ±10% range. Twenty four samples were positive, indicating a prevalence of 46.1% in the studied areas (Canchayllo and Jauja). The kit performed with excellent reproducibility and all individual samples were equally classified by the three laboratories with the exception of two of the samples with readings close to the cut-off, one of them categorized as negative by two of the labs and uncertain by the third one, and another sample classified as (weak) positive by two of the labs and uncertain by the remaining one.

## Concluding Remarks

Two tests were developed and the difference in their performance highlights the importance that careful selection of antibody pairs has to attain a high standard of sensitivity and specificity. The Eg9 test performed with high sensitivity (92.6%) and good specificity (86.4%). These parameters are closely similar to previously reported values for other tests. However, it is very important to keep in mind that the only trustworthy comparison of tests is that obtained with a common panel of samples assayed under identical conditions, which highlights the need of inter-laboratory studies to compare the performance of existing test. The test cross-reactivity was low, and in addition, since it was basically restricted to *T. hydatigena*, most false positive results will still indicate access of dogs to offal. A major contribution of our study is the use of a well-characterized MAb that assures availability and batch-to-batch reproducibility, as well as the formulation of the assay in a kit format with extended shelf life. The Eg9 test is being shared with other control programs in the region and we hope that it will help to monitor the progress of control programs and standardize the epidemiological baseline information in the region.

## Supporting Information

Checklist S1 **STARD Checklist for the Eg9 copro antigen test.**
(DOC)

Flowchart S1 **STARD flow chart detailing the method used to assess the diagnostic performance of the Eg9 copro antigen test.**
(DOCX)
