# Supplementary material for: A Monoclonal Antibody-Based Copro-ELISA Kit for Canine Echinococcosis to Support the PAHO Effort for Hydatid Disease Control in South America
Source: PLoS Negl Trop Dis. 2013 Jan 10;7(1):e1967. doi: 10.1371/journal.pntd.0001967 (PMC3542170; doi:10.1371/journal.pntd.0001967)
Supplement: Flowchart S1 — STARD flow chart detailing the method used to assess the diagnostic performance of the Eg9 copro antigen test. (DOCX) [file pntd.0001967.s002.docx]

**STARD Flowchart**

Number of fecal samples (naturally and Eg experimentally infected dogs) = 108

Number of fecal samples tested by the necropsy reference test = 108

Number positive by the coproELISA = 11

Number also negative by the coproELISA = 70

Number also positive by the coproELISA = 25

Number negative by the coproELISA = 2

Number of dogs positive by the necropsy reference test = 27

Number of dogs negative by the necropsy reference test = 81
